# Supplementary material for: Transmission Cycle of Tick-Borne Infections and Co-Infections, Animal Models and Diseases
Source: Pathogens. 2022 Nov 8;11(11):1309. doi: 10.3390/pathogens11111309 (PMC9696261; doi:10.3390/pathogens11111309)
Supplement: Supplementary file 1 [file pathogens-11-01309-s001.zip › pathogens-1991949-supplementary.pdf]

Supplementary Table S1. TBCI prevalence in engorged ticks in Romania

Pathogens combination	No. of Ticks [a]	Prevalence(%)
Borrelia spp. + Ap + *Rickettsia* spp.	42	18.92
Borrelia spp. + Ap + *R. helvetica* + *Theileria* spp.	2	0.90
Bo. Afzelli + Ap	20	9.01
Bo. garini + Ap	7	3.15
Bo. bavar/ *Bo. garini* + Ap	1	0.45
Bo. Afzelli/B.valaisiana + *Ricketsia* spp	2	0.90
Bo. Myiamotoi + Ap	2	0.90
Bo. bavar/ Bo. Garinii + Ap + Neo.mik	1	0.45
Ap + *R.helvetica*	22	9.91
Ap + *R.monasensis*	7	3.15
Ap + *Theileria* spp.	2	0.90
Ap + *Hepatozoon* spp.	1	0.45
Ba/Bg/*Bo.bavar*/Bmiy + Ap	30	13.51
**Number of ticks analyzed**	**222**	

Abbreviations: *B., Borrelia; R., Rickettsia; Ap, Anaplasma phagocytophilum; Nm, Neoehrlichia mikurensis.* [a] Obtained from supplementary table 1 of Borsan et al.,2021 [28].
